# Learned Immobility Produces Enduring Impairment of the HPA Axis Reactivity in Mice without Replicating the Broad Spectrum of Depressive-Like Phenotype

**DOI:** 10.3390/ijms22020937

**Published:** 2021-01-19

**Authors:** Sébastien Bullich, Sarah Delcourte, Nasser Haddjeri, Bruno P. Guiard

**Affiliations:** 1 Centre de Recherches sur la Cognition Animale (CRCA), Centre de Biologie Intégrative (CBI), Université de Toulouse, CNRS UMR5169, UPS, 31062 Toulouse, France; sebastien.bullich@univ-tlse3.fr; 2 INSERM, Stem Cell and Brain Research Institute U1208, University of Lyon, Université Claude Bernard Lyon 1, 69500 Bron, France; sarah.delcourte@inserm.fr (S.D.); nasser.haddjeri@inserm.fr (N.H.)

**Keywords:** animal model, antidepressant, depression, forced swim stress, HPA axis, serotonin

## Abstract

The forced swim stress test (FST) is widely used for screening pharmacological or non-pharmacological strategies with potential antidepressant activities. Recent data have suggested that repeated FST for five consecutive days (i.e., 5d-RFSS) could be used to generate a robust depressive-like phenotype in mice. However, the face, construct, and predictive validities of 5d-RFSS have been recently challenged. This study took advantage of recent findings showing that mice vulnerability to anxiety is enhanced when animals are stressed during the dark phase, to provide new insight into the relevance of this model. Our results showed a progressive increase in time of immobility in 5d-RFSS mice relative to control non-stressed animals (sham). Three weeks later, we noticed that 5d-RFSS mice injected with the vehicle compound (Veh) still exhibited a high level of immobility in the FST whereas this behavior was reversed by the antidepressant drug amitriptyline (AMI). However, 5d-RFSS/Veh and 5d-RFSS mice/AMI mice showed normal performances in the open field, the novelty suppressed feeding and the tail suspension tests. Despite this lack of generalized behavioral deficits, an impairment of different parameters characterizing the hypothalamic-pituitary-adrenal (HPA) axis reactivity was evidenced in 5d-RFSS mice/Veh but not in 5d-RFSS mice/AMI. Despite anomalies in the HPA axis, the activity of the central serotonergic system remained unaffected in 5d-RFSS mice relative to controls. From our results, it is suggested that learned immobility does not replicate the broad spectrum of depressive symptoms observed in other chronic models of depression such as the unpredictable chronic mild stress (UCMS) model, the chronic social defeat stress (CSDS) model or chronic corticosterone (CORT) exposure but its influence on the HPA axis is remarkable. Further experiments are warranted to makes this model suitable for modelling depression and therefore refine its translational applicability.

## 1. Introduction

Major depression (MD) is a complex disease with heterogeneous symptoms [1]. The source of heterogeneity arises from several factors, including, but not restricted to, the evolution of symptoms over time, gender, age, the presence of comorbidities, the individual sensitivity to treatments, and of course, genetics [2]. On these grounds, modeling MD in animals and, more specifically, in rodents is a major challenge, while this is indispensable to examine neuronal circuits along with cellular and molecular mechanisms regulating emotional states. Animal models are also crucial to screen pharmacological and non-pharmacological antidepressant strategies [3] and, therefore, to refine their translational applicability. 

Based on the observation that a significant proportion of depressed patients display high levels of circulating cortisol [4], the majority of animal models currently available rely on the alteration of the hypothalamic-pituitary-adrenal (HPA) axis. The most common models used in neuropsychopharmacology are the unpredictable chronic mild stress (UCMS), the chronic social defeat stress (CSDS), and the corticosterone (CORT) models. The latter has been widely used, and several pieces of evidence emphasize its face, construct, and predictive validity. For instance, CORT exposure in mice elicits behavioral response recapitulating the core symptoms of MD such as despair, anhedonia, decreased self-care, and anxiety [5,6], as well as impairment of cognitive performances [7], sleep disruption [8], and somatic pain [9]. Interestingly, the chronic administration of CORT also elicits neurobiological impairments associated with MD, notably the fact that it leads to a significant reduction of adult hippocampal neurogenesis [5,10]. Finally, in terms of predictive value, CORT mice are sensitive to treatments that show therapeutic activity in MD [5,11,12]. Despite these results, the chronic exposure to CORT but also UCMS and CSDS has important limitations and pitfalls. Beyond the fact that these models do not allow capturing some important symptoms of MD, such as the feelings of sadness, guilt, or suicidal thoughts, and they are questionable across many other aspects. For example, CORT exposed females are insensitive to this procedure [13], whereas it is well known that the prevalence of MD is twice higher in women than in men [14]. CORT, UCMS, or CSDS are also notoriously known to be cumbersome procedures requiring a regular follow-up of the animals until they harbor a depressive-like phenotype. Finally, there has also been some difficulty in replicating results across laboratories [15], and the individual variability can be another serious impediment. Indeed, there remains a large variance in behavior outcomes since some animals display resistance to stress (resilience), while others are susceptible [16].

These observations have prompted researchers to develop new procedures, and the anhedonia test has drawn attention, notably due to its facility and rapidity of setting up. In this model, mice are forced to swim for 10 min for five consecutive days, and a progressive increase in the time of immobility is observed. Although such behavior can be interpreted as the establishment of resignation, the relevance of the five-day repeated forced swim stress (5d-RFSS) test as a model of depression remains somewhat equivocal. In particular, it has been argued that the high level of immobility observed at the end of the procedure reflects an adaptive learned behavioral response rather than a depressive-like phenotype [17]. In a recent article, Goodyear’s group yielded interesting results showing that C57BL/6J or BALB/cJ mice subjected to the 5d-RFSS test do not display anhedonia evaluated in the sucrose preference test and abnormal level of anxiety or resignation in the open field or tail suspension test, respectively [18]. However, it has been proposed that this lack of effect might arise from the experimental conditions in which animals are tested. In particular, it has been pointed out that a stable and enduring depressive-like state can be unveiled when the 5d-RFSS test is performed during the early part of the dark phase [19,20].

In light of these conflicting results, the present study was aimed to evaluate the validity of the 5d-RFSS test considering the above-mentioned chronobiological aspects. To do so, we stressed the mice during the dark phase, and three weeks later, we conducted a battery of behavioral tests along with electrophysiological and neurochemical experiments to assess the functional activity of the HPA axis and the central serotonergic systems. Moreover, we tested the ability of the antidepressant amitriptyline to reverse the putative anomalies encountered. This set of experiments help us to shed some light on the relevance of such a model to study MD and the efficacy of treatment. 

## 2. Results

We first stressed C57BL/6J mice by forcing them to swim for 10 min once a day during the early dark phase for five consecutive days (Table 1).

### 2.1. Effects of 5d-RFSS on Depressive-Like Behavior in C57BL/6J Mice

**Figure 1:**
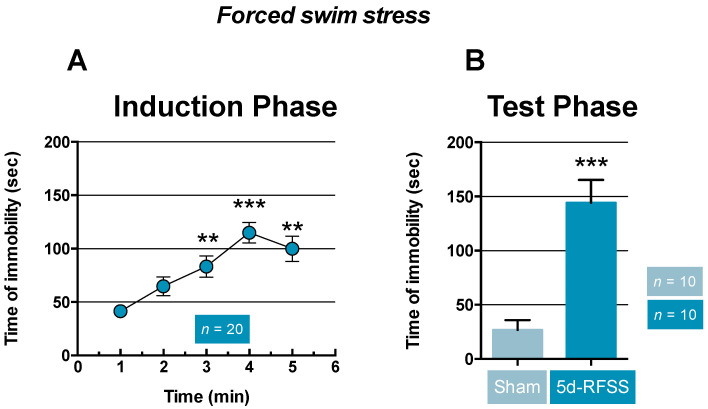
Effects of 5d-RFSS on time of immobility in C57BL/6J mice. (**A**) Data are means ± standard error of the mean (SEM) of time of immobility (sec) in the forced-swim tests (FST) during the induction phase (days 1–5). Statistical analysis: one-way ANOVA with repeated measures indicated significant effects of time (F(4,76) = 13.5; *p* < 0.001). ** *p* < 0.01 and *** *p* < 0.001: significantly different from D1 (*n* = 20) using Fischer’s PLSD post-hoc test. (**B**) Data are means ± SEM of time of immobility time (s) during the test phase in 5d-RFSS mice and their controls (sham) that were not subjected to forced swim during the induction phase. Statistical analysis: Student’s *t*-test, t1, 18 = 5.34. *** *p* < 0.001: significantly different from control non-stressed mice (sham). *n* = 10 mice per group.

As previously reported, we noticed that mice showed a progressive and significant increase in time of immobility from day three to day five relative to day one (*p* < 0.01, *p* < 0.001, and *p* < 0.01; Figure 1A). During the test phase, the time of immobility was significantly higher in C57BL/6J mice that had undergone forced swim stress (i.e., 5d-RFSS mice) compared to control non-stressed animals (sham) (*p* < 0.001; Figure 1B).

After the induction phase, 5d-RFSS mice were split into two groups, one group treated with vehicle (Veh) and the other with the antidepressant drug amitriptyline (AMI) for three weeks (Table 1). The randomization of the animals was based on their performances in the last session of the FST. To do so, we checked that the mean ± standard error of the mean (SEM) was not statistically significant between groups before the initiation of treatments. The test phase aimed at comparing 5d-RFSS/Veh, 5d-RFSS/AMI, and control non-stressed (sham) mice in different paradigms.

**Figure 2:**
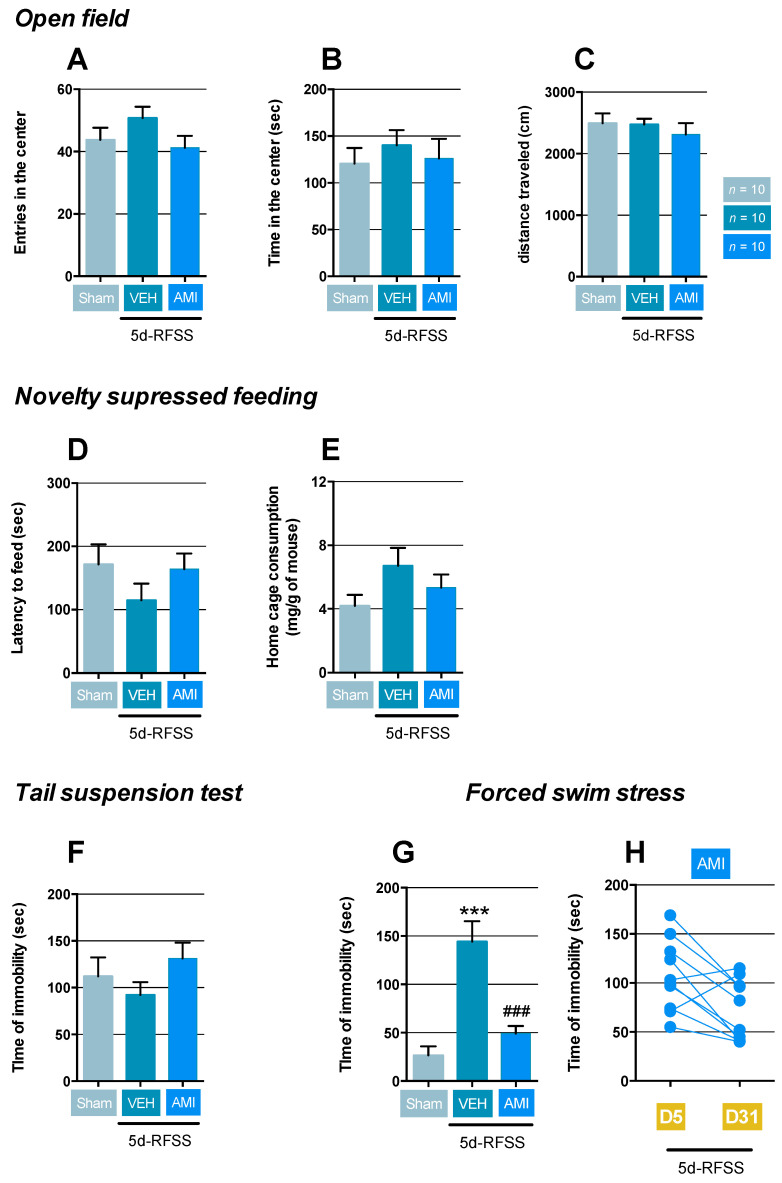
**Behavioral characterization of 5d-RFSS on depressive-like symptoms in C57BL/6J mice.** (**A**–**C**) Data are means ± SEM of number of entries and time spent (in sec) in the center the open field (OF) and, total distance travelled (in cm). One-way ANOVA did not show significant effect of treatment factor for each parameter: (F(2,27) = 1.76; *p* = 0.19), (F(2,27) = 0.3; *p* = 0.73), and (F(2,27) = 0.44; *p* = 0.64), respectively. (**D**–**E**) Data are means ± SEM of latency to feed (in sec) and food consumption (mg/g of mouse) in the novelty suppressed feeding (NSF). One-way ANOVA did not show significant effect of treatment factor for each parameter (F(2,27) = 1.1; *p* = 0.32) and (F(2,27) = 1.9; *p* = 0.16), respectively. (**F**–**G**) Data are means ± SEM of time of immobility (in sec) in the tail suspension test (TST) and forced swim test (FST). One-way ANOVA showed a significant effect of treatment factor for the TST (F(2,27) = 1.29; *p* = 0.28) but a significant effect for the FST (F(2,27) = 21.3; *p* < 0.001). (**H**) Individual data obtained in the 5d-RFSS/AMI group between the induction (D5) and test (D31) phases. *n* = 10 mice per group. *** *p* < 0.001: significantly different from control non-stressed mice (sham). ### *p* < 0.001: significantly different from 5d-RFSS mice using Fischer’s PLSD post-hoc test.

In the open-field (OF), which simultaneously measures novelty-induced exploration and certain components of anxiety, the behavior of 5d-RFSS mice was in a normal range, as evidenced by the lack of differences in the number of entries (*p* > 0.05) and time in the central zone (*p* > 0.05) considered as anxiogenic relative to controls (Figure 2A,B). Similarly, AMI did not modify those parameters compared to control non-stressed mice (*p* > 0.05 and *p* > 0.05; Figure 2A,B). Importantly, locomotor activity was not modified in the experimental groups (Figure 2C). In the NSF in which any delay in food consumption is interpreted as mixed anxiety/depressive-like behaviors, 5d-RFSS mice receiving vehicle or AMI displayed normal latency to feed compared to control (*p* > 0.05 and *p* > 0.05; Figure 2D). No changes in hunger were unveiled in these groups compared to controls since food consumption in the home cage within the 5 min following the test showed no differences (*p* > 0.05 and *p* > 0.05; Figure 2E). These negative results prompted us to use the tail suspension test (TST) and the force swim test (FST) that interrogates resignation, a pure symptom of depression. In the TST, mice are suspended by their tail in order to monitor the escape behavior with immobility time. The latter parameter was not modified in 5d-RFSS/Veh or 5d-RFSS/AMI mice compared to controls (*p* > 0.05 and *p* > 0.05; Figure 2F). In a marked contrast, the time of immobility was significantly increased in 5d-RFSS/Veh submitted to the FST compared to control mice (*p* < 0.001), and AMI reversed this behavioral trait (*p* < 0.001; Figure 2G). Analyzing the individual performance of mice in the FST between the last session of the induction phase (D5) and the test phase (D31), we noticed that 90% of 5d-RFSS/AMI mice showed a decreased time of immobility (Figure 2H). 

### 2.2. Effects of 5d-RFSS on HPA Axis Reactivity in C57BL/6J Mice

**Figure 3:**
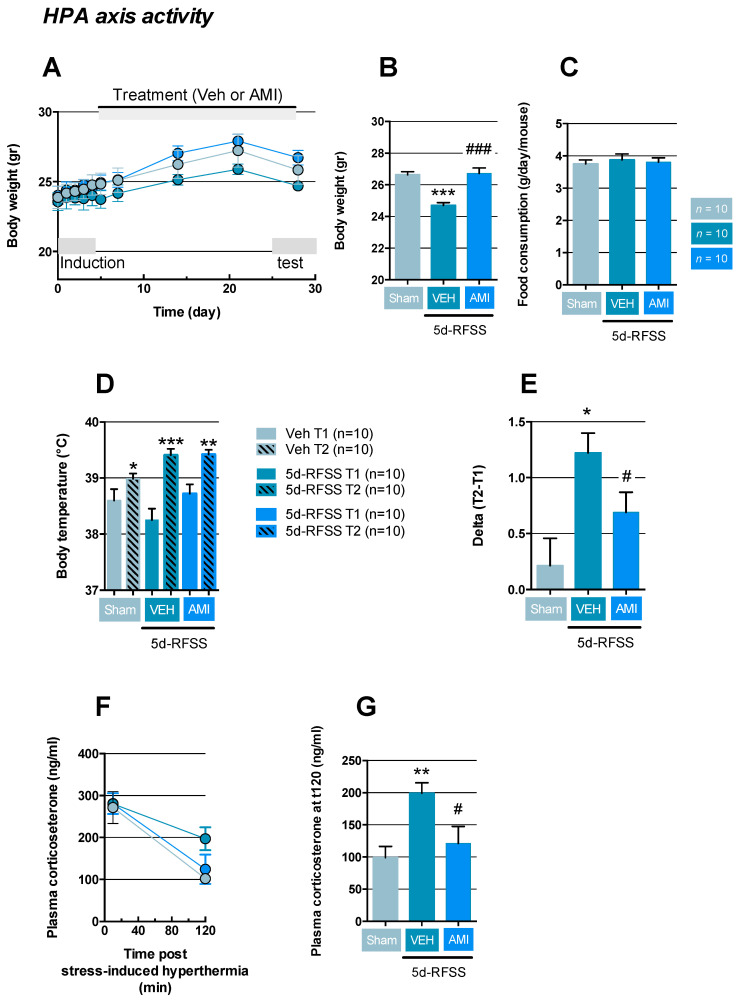
Impact of 5d-RFSS on HPA axis in C57BL/6J mice. (**A**–**C**) Body weight and food consumption. Data are means ± SEM of body weight (in g). Kinetic (**A**,**B**) body weight measured just before the test phase. One-way ANOVA showed a significant effect of treatment factor (F(2,27) = 20.6; *p* < 0.001). Data are means ± SEM of food consumption (C). One-way ANOVA did not show a significant effect of treatment factor (F(2,27) = 0.17; *p* = 0,83]. (**D**,**E**) Stress-induced hyperthermia. Data are means ± SEM of body temperature (in degrees Celsius) after the first (T1) and second (T2) measure (**D**). Two-way ANOVA with repeated measures did not show a significant effect of treatment (F(2,25) = 1.65; p = 0.21) but a significant effect of time (F(1,25) = 48.37; *p* = 0.02) and of the interaction between both factors (F(1,25) = 4.21; *p* < 0.001). Data are means ± SEM of the difference in temperature (in degrees Celsius) between the second and first measure (DelatT=T2-T1) (**E**). One-way ANOVA showed a significant effect of treatment factor (F(2,27) = 6.29; *p* = 0,006) (**F**–**G**) Plasma corticosterone levels. Data are means ± SEM of plasma corticosterone levels (in ng/mL) immediately after stress-induced hyperthermia and 120 min later (**F**). Data are means ± SEM of plasma corticosterone at t120 (in degrees Celsius) (**E**). One-way ANOVA showed a significant effect of treatment factor (F(2,27) = 6.34; *p* = 0.005). *n* = 10 mice per group. * *p* < 0.05, ** *p* < 0.01 and *** *p* < 0.001: significantly different from control non-stressed mice (sham). # *p* < 0.05 and ### *p* < 0.001: significantly different from 5d-RFSS mice using a Fischer’s PLSD post-hoc test.

In agreement with previous data [18], we noticed that five consecutive days of forced swim had no immediate effect on body weight in 5d-RFSS mice compared to controls (Figure 3A). However, after this period of repeated stress, 5d-RFSS/Veh mice showed a lower increase in body weight compared to control non-stressed animals (*p* < 0.001), while this effect was prevented by AMI (*p* < 0.001; Figure 3A,B). Importantly, comparing food consumption between 5d-RFSS/Veh and 5d-RFSS/AMI mice with controls, we did not notice any significant differences between groups (*p* > 0.05 and *p* > 0.05; Figure 3C). Because the latter results could be a marker of HPA dysregulation, we next assessed the neuroendocrine reactivity of this axis using the stress-induced hyperthermia (SIH) test. This paradigm is based on the fact that when mice are confronted with a stressful situation, their body temperature rises. In this paradigm, a first rectal temperature measurement (T1) was performed, followed 10 min later by a second temperature measurement (T2). As expected, we reported that controls (*p* < 0.05), 5d-RFSS/Veh (*p* < 0.001), and 5d-RFSS/AMI mice (*p* < 0.01) displayed hyperthermia after this acute stress (Figure 3D). Since previous data demonstrated that the higher the DeltaT (T2–T1), the more stressed are the mice [21], we calculated this parameter in each group. DeltaT was significantly higher in 5d-RFSS/Veh compared to non-stressed animals (*p* < 0.05), whereas such response was significantly attenuated in 5d-RFSS/AMI (*p* < 0.05; Figure 3E). Blood was collected in each group immediately and 120 min after the stress-induced hyperthermia test. This procedure allowed us to assess plasma corticosterone concentrations post-stress and recovery capabilities. Our results revealed that all groups displayed similar levels of plasma corticosterone following the test (Figure 3F). However, when measuring the corticosterone 120 min after acute stress as a proxy for HPA negative feedback effectiveness, our results showed that the plasma levels of this hormone were significantly higher 5d-RFSS/Veh mice compared to control non-stressed mice (*p* < 0.01), suggesting lower negative feedback effectiveness in the former group and this was reversed in 5d-RFSS/AMI (*p* < 0.05; Figure 3G).

### 2.3. Effects of 5d-RFSS on Serotonergic Neurotransmission in C57BL/6J Mice

**Figure 4:**
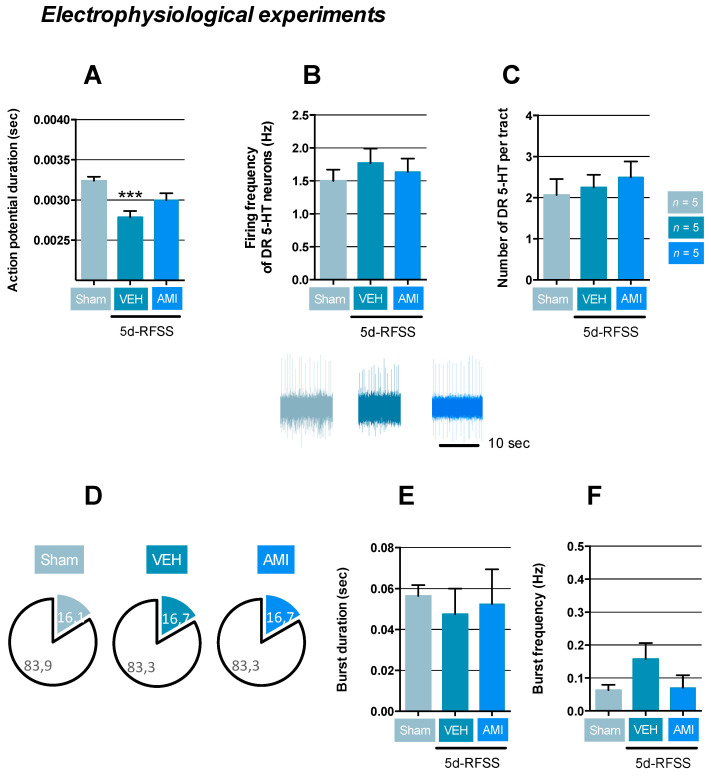
Impact of 5d-RFSS on the activity of dorsal raphe (DR) 5-HT neuronal activity in C57BL/6J mice. (**A**–**C**) Data are means ± SEM of the action potential duration (sec), the firing rate (in Hz), and the number of DR 5-HT neurons recorded per tract using extracellular in vivo electrophysiology. One-way ANOVA showed a significant effect of treatment factor on action potential duration (F(2,95) = 10.17; *p* < 0.001) but not on the firing (**B**) (F(2,95) = 0.48; *p* = 0.61) and the number of cells encountered (F(2,40) = 0.34; *p* = 0.71). Examples of typical recordings DR 5-HT neurons obtain in control non-stressed (sham), 5-dRFSS/Veh, and 5d-RFSS/AMI are also depicted. *** *p* < 0.001: significantly different from control non-stressed mice (sham) using Fischer’s PLSD post-hoc test. *n* = 5 mice per group. (**D**–**F**) Electrophysiological properties of DR 5-HT neurons displaying burst activity. Percent of DR 5-HT neurons discharging in bursst (**D**). Data are means ± SEM of burst duration (s) (**E**) and frequency (Hz) (**F**). One-way ANOVA did not show significant effects of treatment factor on these parameters (F(2,12) = 0.29; *p* = 0.82] and (F(2,12) = 1.39; *p* = 0.28), respectively.

Because previous evidence suggests that modification in the HPA interferes with the serotonergic system and vice-versa [22], we next sought to determine the activity of the serotonergic raphe-hippocampal neurocircuit in 5d-RFSS/Veh and 5d-RFSS/AMI compared to control non-stressed animals. Electrophysiological experiments indicated that mice that had undergone 5d-RFSS showed a significant reduction of their action potential duration compared to controls (*p* < 0.001; Figure 4A). However, this modification did not influence dorsal raphe (DR) serotonin (5-HT) neuronal activity as evidenced by a normal firing frequency and the number of spontaneously active neurons recorded per tract (*p* > 0.05 and *p* > 0.05; Figure 4B,C). AMI did not modify these parameters neither (*p* > 0.05, *p* > 0.05 and *p* > 0.05; Figure 4A–C). Since some DR 5-HT neurons displayed a bursting activity, we also studied the properties of this subpopulation. As previously reported, we found that approximately 16% of DR 5-HT neurons had a spontaneous burst-type firing pattern composed of two action potentials (i.e., doublets) in all experimental groups. However, the characteristics of these bursts in terms of duration and frequency were not modified in 5d-RFSS/Veh (*p* > 0.05 and *p* > 0.05) and 5d-RFSS/AMI (*p* > 0.05 and *p* > 0.05) compared to control non-stressed animals (Figure 4D,E). 

Importantly, the lack of apparent effect of AMI does not necessarily underlie an absence of response. Indeed, it is well documented that serotonergic antidepressant drugs exert a time-dependent effect on DR 5-HT neuronal activity. In support of this assertion, we showed in non-stressed and 5d-RFSS animals that a two days’ treatment with AMI resulted in a 65% ± 7% (*p* < 0.01) and 59% ± 5% (*p* < 0.01) decrease in DR 5-HT neuronal firing rate relative to the corresponding control groups. Together, our findings indicated that the firing rate of DR 5-HT neurons is decreased after short-term administration of AMI whereas a gradual recovery of firing rate to basal is observed after prolonged exposure, likely due to progressive desensitization of somatodendritic serotonin 1A receptor subtype (5-HT1A) [23].

**Figure 5:**
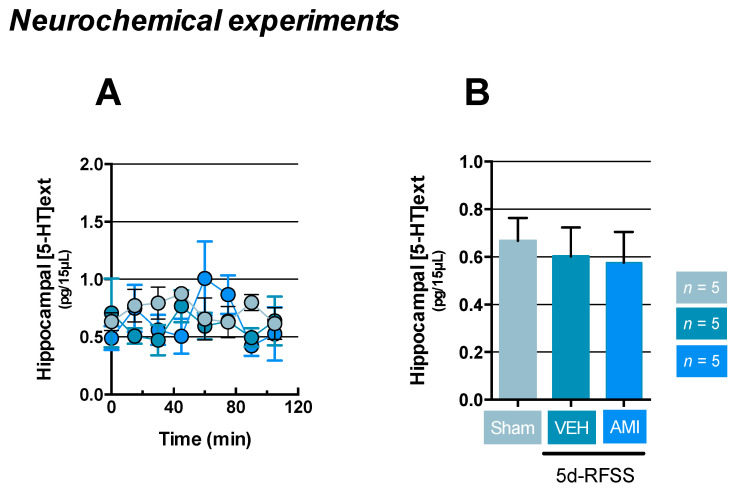
Impact of 5d-RFSS on the hippocampal extracellular 5-HT levels in C57BL/6J mice. (**A**,**B**) Intracerebral microdialysis experiments. Time course of the amount of 5-HT outflow collected during 0–120 min expressed in pg/15 µL (**A**). Data are means ± SEM of the amount of hippocampal 5-HT outflow. One-way ANOVA did not show significant effect of treatment factor (F(2,12) = 0.13; *p* = 0.87] (**B**). *n* = 5 mice per group.

Finally, given that the degree of neurotransmission could be dependent or independent of changes in firing rate, we then measured the extracellular hippocampal 5-HT levels ((5-HT)ext) in our experimental groups. Results indicated that neither 5d-RFSS/Veh (*p* > 0.05) nor 5d-RFSS/AMI mice (*p* > 0.05) displayed changes in (5-HT)ext relative to control non-stressed mice (Figure 5A,B).

## 3. Discussion

Disrupted circadian rhythms are a core feature of many psychiatric conditions [24], and it has been shown that mice selectively bred for high anxiety- and depression-like behaviors exhibit aberrant circadian rhythms [25]. Moreover, comparing the anxiety/depressive-like phenotype of rats previously exposed to different dark/light (LD) cycles, it was found that the free running period under dark conditions was associated with anxiety-like behaviors when animals were tested during the light phase [26]. The latter results strongly suggest a relationship between circadian locomotor parameters and mood-related behaviors in healthy animals. In light of these findings, the present study challenged the validity of the 5d-RFSS in C57BL/6J, taking into consideration the critical factor of the time of the day at which the sessions of swim stress were conducted.

One of the most remarkable results reported herein is the fact that 5d-RFSS procedure under dark conditions induced a progressive increase in time of immobility in the FST during the induction phase. Moreover, and as previously reported [18,19,27], this effect persisted for at least three weeks as evidenced by a significantly higher time of immobility in 5d-RFSS mice than in control non-stressed animals (sham). Because it is unlikely that any one test accurately replicates the ensemble of complex phenotypes seen in depression, we conducted additional experiments in order to determine whether this behavior reflects an enduring anxiety/depressive-like state. Animals were thus subjected to other paradigms, but we noticed that 5d-RFSS/Veh mice had a normal level of innate anxiety. This was supported by the observation that the number of entries and time spent in the central zone of the OF or latency to feed in the NSF was not modified in 5d-RFSS/Veh mice. Assessing another core symptom of depression (i.e., resignation), we showed in 5d-RFSS/Veh mice subjected to the TST that the time of immobility resembled those of controls, whereas this parameter was significantly increased in the FST. These results suggest that C57BL/6J mice did not develop robust neurobehavioral abnormalities, thereby confirming initial studies reporting that 5d-RFSS mice had normal hedonic and resignation behaviors during the test phase [18]. Besides the assessment of the anxiety/depressive-like phenotype of 5d-RFSS/Veh mice, we decided to test the effects of the non-selective monoamine reuptake inhibitor amitriptyline (AMI). The choice of this antidepressant drug relied on its ability to promote antidepressant-like effects after systemic administrations of low doses not only in naïve mice [28] but also in relevant animal models of depression [29]. AMI did not promote anxiolytic/antidepressant-like responses in the OF, NSF, and TST. Such results can be explained by the fact that 5d-RFSS procedure had no effect in those behavioral tests, although, there is precedence in the literature showing that antidepressant drugs may elicit beneficial effects in non-pathological conditions. However, in the FST, we observed that AMI reversed the 5d-RFSS-induced increase in the time of immobility during the test phase. Such phenotype in 5d-RFSS/Veh and 5d-RFSS/AMI, cannot be attributable to a sedative or psychostimulant effect since the distance traveled by the mice in these groups of mice in the OF was comparable to that of controls. 

Consequently, the fact that we stressed the mice under the dark conditions did not allow providing a more robust phenotype than that described earlier [18]. It is possible that other experimental parameters account for the mitigated phenotype reported in this study. For instance, in addition to stress the animal under dark conditions, Delcourte and collaborators applied a new swim stress session each week before the test phase [20]. Such recall could be a pre-requisite to obtain behavioral anomalies in different paradigms. In the present study; however, we can fairly interrogate the effects observed in the FST during the test phase not only on the ability of the 5d-RFSS test to induce a pro-depressive phenotype but also on that of AMI to exert presumed antidepressant-like effects. Indeed, it has been emphasized that repeated forced-swim sessions could induce a learned behavioral response [17]. This hypothesis is further supported by the lack of effect in the TST, which probes the same behavior as the FST but in which mice were tested only once. If this hypothesis of learned behavioral response in the FST is correct, we can then assume that the ability of AMI to decrease the time of immobility could, on the contrary, reflect an impairment of memory. In this way, 5d-RFSS/AMI mice could behave like their first experience in the FST. The majority of preclinical studies indicate that serotonin and/or norepinephrine reuptake inhibitors exert beneficial effects on cognitive performances, notably in relation to their positive influence on brain plasticity in brain regions such as the frontal cortex and the hippocampus [30]. However, one would bear in mind that AMI, as the other tricyclics, could produce opposite effects. Although a limited number of studies have been implemented to test this antidepressant drug on memory, there is evidence that its administration immediately after inhibitory avoidance training impaired memory consolidation [31]. In keeping with the latter results, AMI was also shown to potentiate scopolamine-induced memory deficit in mice. Such a negative impact on cognitive performances was attributed to the anticholinergic properties of AMI, which binds and blocks muscarinic/nicotinic receptors [29,32]. On this ground, it is not surprising that AMI was shown to impair memory in depressed patients [33]. 

In an attempt to further characterize 5d-RFSS/Veh mice, we then conducted experiments aimed at evaluating the activity of the HPA axis. We first examined body weight, which is usually lowered in depressed patients [34] or in relevant animal models such as UCMS [35] or CSDS [36]. Interestingly, although a progressive increase in body weight was observed in all experimental groups during the protocol, this increase was less pronounced in 5d-RFSS/Veh mice, whereas AMI reversed this effect. The influence of HPA axis and body weight is not fully understood, but it has been proposed that stress might promote a reduction in the number of orexins neurons [37] and/or the expression of orexin receptors subtype in the hypothalamus [38], thereby impacting food intake and subsequently body weight. However, we noticed that 5d-RFSS/Veh and 5d-RFSS/AMI had normal eating behavior reinforcing the hypothesis that the lower body weight in 5d-RFSS/Veh reflects a defect in the emotional circuit of the HPA axis. To confirm this hypothesis, the HPA axis was then probed using the stress-induced hyperthermia (SIH) test. In all groups, an increase in body temperature was observed in response to the manipulation of the animals, notably between the first and second measurements. These results agree with previous findings showing that animals exposed to chronic stress exhibit an enhanced hyperthermic response to a novel stress [39], and this response is seemingly driven by the HPA axis. Indeed, it was reported that the peak of hyperthermia coincides with the peak of plasma corticosterone levels and c-fos immunoreactivity in corticotropin-releasinghormone (CRH)-containing neurons of the paraventricular hypothalamic nucleus [40]. Interestingly, such an increase in body temperature was higher in 5d-RFSS/Veh than in control non-stressed mice and restored in 5d-RFSS/AMI mice. These findings provided more experimental evidence that AMI was able to counteract 5d-RFSS-induced HPA axis dysfunction. Such effects in the SIH were already reported with anxiolytics (i.e., benzodiazepines) and serotonergic antidepressant drugs [41,42]. Going one step further, we then sought to measure plasma corticosterone levels at different time points. We noticed that this parameter was similar between groups immediately after the SIH. However, 5d-RFSS/Veh mice displayed blunted HPA axis inhibitory feedback since a lower decrease in plasma corticosterone levels were highlighted 2 h the acute stress of taking body temperature compared to control non-stressed animals. Again, AMI reversed this effect, as shown by the complete recovery of the inhibitory feedback process in this group of mice. Although the mechanisms underpinning the effects of 5d-RFSS and those of AMI have yet to be determined, a recent study shows that the manipulation of adult hippocampal neurogenesis influence the negative feedback of the HPA in UCMS subjected mice [43]. Further experiments will help better understand the anatomical and functional interactions between the hippocampus and the HPA axis and how the 5d-RFSS procedure might produce enduring effects on related neuronal circuits.

Because several lines of evidence have suggested that HPA axis has a critical role in the regulation of serotonergic neurotransmission [22], we then tested whether 5d-RFSS negatively reverberated on serotonergic tone in the hippocampus. Unfortunately, we did not detect any changes in the electrophysiological and neurochemical parameters evaluating the activity of the serotonergic system. In particular, we showed that 5d-RFSS/Veh mice did not show any modifications of DR 5-HT neuronal activity. Indeed, although action potential duration was decreased in 5d-RFSS/Veh mice, this modification did not reverberate of 5-HT neuron activity as evidenced by the normal pattern of discharge of neurons discharging with single spikes or bursts. It is plausible that the delay between the stress procedure and the electrophysiological analysis accounted for such negative effects since we have recently shown decreases in the firing rate of DR 5-HT neurons in the CORT model, but under these conditions, mice were receiving the stress hormone for the whole duration of the protocol [44]. Unfortunately, there are no studies evaluating the firing rate of DR 5-HT neurons after UCMS or CSDS. This would have helped us confirm the latter hypothesis. Interestingly, 5d-RFSS/AMI mice did not show changes in DR 5-HT neuronal activity either. At first glance, this is not surprising since the chronic administration of serotonergic antidepressant drugs does not increase the serotonergic firing rate. Indeed, electrophysiological studies have shown that acute and short-term administration of serotonergic antidepressant drugs, including tricyclics, reduces the firing activity of DR 5-HT neurons. However, as the treatment is pursued, there is a complete recovery of their firing activity due to the desensitization of the somatodendritic 5-HT1A autoreceptors, which control their firing activity [45]. In general, such loss of function of 5-HT1A autoreceptors in the DR is accompanied by a significant increase in extracellular 5-HT levels [46]. In agreement with electrophysiological data, the basal hippocampal extracellular 5-HT levels were not modified in 5d-RFSS/Veh and 5d-RFSS/AMI. Nevertheless, given that AMI blocked the serotonin transporter (SERT) and norepinephrine transporter (NET) for three weeks, one would expect that such property resulted in a significant increase in extracellular 5-HT levels at the nerve terminals. It was proposed that selective serotonin reuptake inhibitors (SSRI) or serotonin/norepinephrine reuptake inhibitors (SNRIs) may not be homogeneous with regard to their ability to enhance 5-HT tone, but to our knowledge, there is no study investigating the neurochemical effects of AMI on extracellular 5-HT levels. However, several hypotheses may be proposed to explain the lack of effect of AMI. Because 5-HT outflow measured by microdialysis reflects a balance between 5-HT uptake and release, we assumed that any increase in 5-HT release at the nerve terminals might be counterbalanced by an active 5-HT degradation process through an increased monoamine oxidase expression and/or activity. Alternative processes might have occurred targeting other mechanisms involved in the auto- and/or hetero-regulation of the 5-HT system. Finally, it would have been interesting to determine whether or not the chronic administration of AMI impacted the extracellular norepinephrine levels in the hippocampus or other brain regions involved in the regulation of emotional states. 

Overall, our results indicate that 5d-RFSS without any recall session every week did not induce conclusive emotional impairments in male C57BL/6J mice even if the stress procedure was implemented during the dark phase. Although a presumed pro-depressive like phenotype was highlighted in the FST paradigm, the fact that such phenotype was restricted to this test questions its face validity. Instead, a learned behavioral response seems more likely to occur, and this was strengthened by the ability of AMI—known to exert negative effects on memory—to reverse the 5d-RFSS-induced increase in time of immobility. Consequently, we could also challenge the predictive validity, but this point requires additional experiments. In particular, we need to determine if antidepressant drugs without influence on cognitive performances increase the time of immobility in the FST during the test phase. It would also be interesting to tackle the question of gender in this paradigm and determine whether females are more prone to develop anxiety/depressive-like symptoms than males. Such experiments will help us determining whether the 5d-RFSS is appropriate to identify novel antidepressant strategies but also if it could be considered as an animal model of treatment resistance in males and/or females. 

## 4. Materials and Methods

### 4.1. Animals

Seven weeks old adult C57Bl/6J male mice were purchased from Janvier Laboratories (Le Genest-Saint-Isle, France) and housed five per cage, with a 12/12 h day/light cycle (light on at 8:00 a.m). Food and water were available ad libitum. All experimental procedures were conducted in accordance with the European directive 2010/63/EU and were approved ‘05/02/2019) by the French Ministry of Research and the local ethics committee (APFIS # 2018100110245946#16913).

### 4.2. Drugs

Amitriptyline hydrochloride (AMI, Sigma-Aldrich, Saint-Quentin Fallavier, France) was dissolved in tap water solution and delivered in the drinking water at the dose of 6 mg/kg for three weeks in opaque bottles. This dose was chosen for its ability to efficiently cross the blood brain barrier [47] and to produce antidepressant-like effects after acute administration [28]. The dose was adjusted according to the weekly consumption of mice, and tap water was used as the vehicle. Solutions were changed twice a week. Mice continued to drink AMI throughout the duration of behavioral testing. 

### 4.3. The Five Days Repeated-Forced Swim Stress and Experimental Protocol

Stressed mice were forced to swim in a cylindric container (diameter, 20 cm; height, 30 cm) containing water (23 ± 2 °C) for five consecutive days named the induction phase (Table 1) as previously described [18]. Importantly, each session of the forced stress lasted 10 min and was conducted during the dark phase when mice are active as recommended [20]. On day five, after the swim stress, mice were split into two groups, one receiving a continuous administration of amitriptyline in the drinking water and the other its vehicle for three weeks. The test phase (Table 1) started on day 28 during which mice were successively tested in the open field (OF), the novelty suppressed feeding (NSF), the tail suspension test (TST), and the forced swim test (FST). Control mice were not subjected to forced swim on either day during the induction phase. These non-stressed mice were phenotyped only during the test phase. All behavioral tests were performed in the morning to avoid differences in locomotor activity, and other variables affected by circadian rhythm and mice performances were evaluated from the least to the most stressful test, thereby decreasing the chance that one test might affect the behavior evaluated in the subsequent paradigm. After these tests, all mice were subjected to the stress-induced hyperthermia test before being tested in electrophysiological and neurochemical experiments (Table 1).

### 4.4. Behavioral Paradigms 

#### 4.4.1. The Open-Field 

Mice were allowed to explore a circular arena (diameter: 40 cm) for 10 min in a dark room without any remarkable features. A digital camera was placed above the device to record each session. For this video tracking, Noldus software (Ethovision, Wageningen, The Netherlands) was used to measure the time spent and the number of entries in the center. The total distance traveled was also recorded to make sure that mice from the different groups did not experience psychostimulant or sedative effects. 

#### 4.4.2. The Novelty-Suppressed Feeding

Mice were fasted for 24 h prior to the test. Then they were then placed in a new box (30 × 60 cm) containing in its center a single food pellet on a white filter paper platform. The pellet was exposed to intense lighting (60 W) in order to reinforce the anxiogenic nature of the device. Latency to feed was measured with a cut-off time of 10 min. Importantly, to ovoid putative bias linked to the perceived odors from the preceding congener, the sawdust was changed after each run. At the end of the test, mice returned to their home cage, and food consumption was quantified for 5 min to verify the absence of differences in hunger and/or motivation.

#### 4.4.3. The Tail Suspension Test 

Mice were suspended by their tails with tape to avoid any contact on to nearby sur- faces. The test time of immobility was manually scored as an index of resignation by a competent experimenter for 6 min.

#### 4.4.4. The Forced-Swim Stress 

Briefly, mice were individually placed in a beaker (height: 23 cm; diameter: 20 cm) containing 15 cm of water (23 ± 2 °C) for 6 min. The process was videotaped, and the dependent variable was the time of immobility calculated (during the induction and test phases) for the last 4 min as previously recommended [48]. These experiments were scored by an experienced observer who did not know the treatment. Floating or only slight movement to maintain balance was considered immobility.

#### 4.4.5. Body Weight and Food Consumption Measurements 

All experimental groups were body weight matched at the onset of the experiments. Body weights were noticed every day during the induction phase, then once a week during the treatment period, and finally, at the end of the test phase. Regarding food consumption, we calculated the quantity of food intake (in g/day/mouse). This value was extrapolated from each cage containing five animals at a specific time point. We then calculated food consumption as the average food intake using different time points from the beginning of the treatment period to the end of the test phase. 

#### 4.4.6. The Stress-Induced Hyperthermia Test 

In the SIH, mice in their home cages were moved to the testing room and allowed to acclimate for 1 h (between 8:30 and 9:30 am). Animals were kept in groups for the assessment of intrarectal body temperature using a lubricated probe inserted approximately 2 cm deep (BIO-BRET 3, Bioseb, Vitrolles, France). In our procedure, stress-induced hyperthermia was determined as the difference between temperature 2 and temperature 1 (T2–T1) by respecting an interval of 10 min between both measures.

### 4.5. Plasma Corticosterone Levels

Blood was collected from the tip of the tail vein. It was immediately centrifugated after collection (5000 rpm for 10 min) to extract plasma. This fraction was stored at −20 °C until analyzed for total corticosterone levels using an enzyme-linked immunosorbent assay (ELISA) Kit (Enzo Life Sciences (ELS) AG, Villeurbanne, France).

### 4.6. In Vivo Single unit Recordings of 5-HT Neurons in the Dorsal Raphe 

After the behavioral phenotyping, half of the mice were anesthetized with choral hydrate (400mg/kg; ip). Once asleep, they were positioned in a stereotaxic frame. Single cell recordings of serotonergic neuron located in the dorsal raphe (DR) were assessed using a glass micropipette (Stoelting, Wood Dale, IL, USA) prepared from a gravitational puller (Narishige, London, United-Kingdom) and filled with a 2 M NaCl solution. After checking that their impedances ranged between 2.5 to 5 MHom, pipettes were inserted in the DR using the following coordinated: 0.2–0.5 mm posterior to the interaural line on the midline and lowered at 3.5 mm depth from the brain surface. Serotonergic neurons were identified according to two main criteria: a regular firing rate between 0.5 to 2.5 Hz and a long-duration positive action potential. Multiple tracks were assessed to monitor the average discharge frequency of DR 5-HT neurons for each mouse as well as the number of neurons recorded per track was also determined as previously done in our lab [49].

### 4.7. In Vivo Intracerebral Microdialysis in the Hippocampus of Freely Moving Mice

The other half of the mice were anesthetized with choral hydrate injection (400 mg/kg; intraperitoneal injection (ip)) and then positioned in a stereotaxic frame to implant a microdialysis probe (effective membrane length 2.0 mm) in the ventral hippocampus. According to the mouse brain atlas [50], the coordinates of implantation from bregma were: anteroposterior (AP): 2.7; lateral (L): ±2.7; and ventral (V): 3.5 mm. One day after, the home-made probes were connected to a microinjection pump providing continuous perfusion (flow rate: 1.5 μL/min) of artificial cerebrospinal fluid. The first 2 h were used to stabilize the exchange between the interstitial fluid and the probe. After this stabilization period, microdialysates were collected every 15 min for a 2 h period, and each of the microdialysates were analyzed by HPLC (XL-ODS, 4.6 × 7.0 mm, particle size 3 μm; Beckman Coulter, Palo Alto, CA, USA), associated with an amperometric detector (1049A, Hewlett-Packard, Les Ulis, France). Area under the curve (AUC) values for the extracellular 5-HT levels ((5-HT)ext) were calculated as a percent of the baseline for each sample. The sensitivity limit for 5-HT was ~0.5 fmol sample 1 (signal-to-noise ratio = 2).

### 4.8. Statistical Analysis

In the present manuscript, results were expressed as mean ± SEM values. A one-way ANOVA using time as the main factor was performed for Figure 1A. A one-way ANOVA using treatment as the main factor was performed for Figure 2A–G, Figure 3B,C,E,G, Figure 4A–C,E,F, and Figure 5B. When the main effects were significant, treatment comparisons were analyzed using a Fischer’s PLSD (Least Significant Difference) post-hoc test. The Student’s *t*-test was applied to compare immobility time between the two experimental groups depicted in Figure 1B. Finally, we assessed a two-way ANOVA with repeated measures for the body temperature (Figure 3D). The threshold for significant differences was set at 0.05. All analyses were performed using GraphPad Prism software (v6.0h, San Diego, CA, USA).

## Figures and Tables

**Table 1:** Experimental protocol of 5d-RFSS.

		Control Non-stressed Animals (Sham)	5d-RFSS/Veh	5d-RFSS/AMI
**Induction Phase** Swim stress	D1		●	●
	D2		●	●
	D3		●	●
	D4		●	●
	D5		●	●
Treatments for three weeks	D5-D26	Veh	Veh	AMI
**Test Phase**	OF (D26)	●	●	●
	NSF (D28)	●	●	●
	TST (D30)	●	●	●
	FST (D32)	●	●	●
HPA axis	SIH	●	●	●
	CORT	●	●	●
Serotonergic system	Electrophysiology	●	●	●
	Microdialysis	●	●	●

Detail of the experimental protocol. C57BL/6J mice were subjected to forced swim stress 10 min for five consecutive days (5d-RFSS) while control animals were kept in their home cage (induction phase). After this period, 5d-RFSS were split into two groups receiving either amitriptyline (6 mg/kg) in the drinking water (5d-RFSS/AMI) or its vehicle (tap water: 5d-RFSS/Veh) for 21 days. Control non-stressed animals were not forced to swim during the induction phase and received tap water. After treatments, control, 5d-RFSS/Veh, and 5d-RFSS/AMI mice were subjected to a battery of behavioral tests (Test phase) conducted on days 28 to 33, including the open field (OF), the novelty suppressed feeding (NSF), the forced swim tests (FST). The HPA axis was tested using stress-induced hyperthermia (SIH) and the analysis of plasma corticosterone (CORT). At the end of this period, a part of them was tested in electrophysiological experiments and another part in intracerebral microdialysis to assess the functional status of the serotonergic (5-HT) system. D: day; 5d-RFSS: five-day repeated forced swim stress; Veh: vehicle: AMI: amitriptyline; OF: open-field; NSF: novelty suppressed feeding; TST: tail suspension test; FST: forced swim test; SIH: stress-induced hyperthermia; CORT: corticosterone; HPA: hypothalamic-pituitary-adrenal. Black dots indicate the tests performed in each experimental groups.

## Abbreviations

5d-RFSS	five-day repeated forced-swim stress
AMI	amitriptyline
CSDS	chronic social defeat stress
CORT	corticosterone
DR	dorsal raphe
FST	forced swim stress
HPA	hypothalamic-pituitary-adrenal axis
MD	major depression
NSF	novelty suppressed feeding
OF	open-field
SIH	stress-induced hyperthermia
UCMS	unpredictable chronic mild stress
Veh	vehicle
